# Preconception fear of childbirth: experiences and needs of women fearing childbirth before first pregnancy

**DOI:** 10.1186/s12978-022-01512-9

**Published:** 2022-10-28

**Authors:** Elisabet Rondung, Susanna Magnusson, Elin Ternström

**Affiliations:** 1 Department of Psychology and Social Work, Mid Sweden University, 831 25 Östersund, Sweden; 2 Institution for Health and Welfare, Dalarna University, Falun, Sweden; 3 Department of Women’s and Children’s Health, Uppsala University, Akademiska Sjukhuset, 751 85 Uppsala, Sweden

**Keywords:** Fear of childbirth, Preconception care, Pregnancy, Birth, Maternity care, Women’s experiences

## Abstract

**Background:**

Although early case studies have indicated that fear of childbirth can predate a woman’s first pregnancy, the concept of preconception fear of childbirth is largely unexplored. The few studies reporting on the prevalence of preconception fear of childbirth found higher levels than most prevalence estimates in pregnant populations. However, little is known about women’s fear of childbirth before becoming pregnant. The aim of this qualitative study was to give voice to the experiences of this often-neglected group of women.

**Methods:**

To address the experiences and needs of women who do not dare become pregnant due to fear of childbirth, we conducted nine qualitative interviews and analyzed these using reflexive thematic analysis.

**Results:**

The women perceived childbirth as an extremely risky event and doubted their abilities to cope with it. With increasing age, the fear became more real. It was associated with thoughts of becoming too old to be able to conceive. The women did their best to cope with fear on their own by seeking information, trying not to think about it, and using multiple strategies to avoid becoming pregnant. Despite expressing a strong wish for professional support, they all described very limited opportunities to receive support from maternal care services. They felt abandoned, left on their own in a stressful and constantly ongoing negotiation with themselves, feeling the pressure to decide whether to dare become pregnant or not.

**Conclusion:**

In this study, women expressed having experienced fear of childbirth long before a first pregnancy. They felt abandoned as they had to deal with their fear by themselves, without support from maternal care services. The results point to the necessity of an increased awareness of preconception fear of childbirth. We encourage maternal care services to consider their opportunities to support these women.

## Plain English summary

Many researchers have studied the experiences of pregnant women who are afraid of giving birth. Although it is known that women can fear childbirth long before becoming pregnant, little research has investigated this issue. In this study, we interviewed nine women who wanted to have children but did not dare become pregnant because they were afraid of giving birth. The women perceived childbirth as an extremely risky event and doubted their abilities to cope with it. They tried to cope with their fear by seeking information about pregnancy and childbirth. They also tried not to think about these issues and did what they could to avoid becoming pregnant. The women felt abandoned as they had to deal with their fear on their own. They wanted support from maternal care services, but this was seldom the case. Instead, they felt pressured to decide whether to dare become pregnant or not without support. Here, we want to give voice to the experiences of this often-neglected group of women and make researchers, policy makers, and health care personnel aware of the needs of women who are afraid of giving birth before becoming pregnant. We encourage maternal care services to consider how they can support these women.

## Background

Over the last few decades, fear of childbirth has been increasingly acknowledged in media, clinical care, and research. Although early case studies have identified preconception childbirth fear (i.e., fear of childbirth before a woman’s first pregnancy) [1], almost all research initiatives have focused on pregnant women. However, some exceptions confirm the occurrence of fear of childbirth among women who have never been pregnant. In European, North American, and Australian studies, approximately 26–27% of non-pregnant female students have reported elevated levels of fear of childbirth [2–4]. Interestingly, this figure is higher than most prevalence estimates in pregnant populations, where a pooled prevalence of 14% (range 3.7–43%) has been reported [5].

To date, there is a knowledge gap about preconception fear of childbirth as this issue has been examined in only a handful of published articles, many of them referencing a Canadian research initiative investigating attitudes of university students about pregnancy and giving birth [6, 7]. In the only qualitative study found, content analysis of women’s written comments about labor and birth showed that non-pregnant female students with high levels of fear of childbirth generally perceived birth as an inherently risky and uncontrollable process [7]. In this study from the Canadian project, the women expressed fear of pain, bodily harm, birth injuries, or even death as well as fear of the unknown, fear of feeling exposed or humiliated, and fear of panicking or losing control. Many expressed doubt in their own abilities to cope with labor and birth and a preference for caesarean section (CS) [7]. In many ways, these fears are similar to those expressed by pregnant women [8–11].

In the absence of other qualitative studies, the current knowledge on preconception fear of childbirth is mainly based on quantitative investigations. In the Canadian project, reported levels of fear of childbirth were highest among students who reported that their attitudes about pregnancy and birth largely had been shaped by media. They were also higher in Asian than in Caucasian students [6]. In addition, both Canadian and Australian data has showed that when thinking about giving birth, students with high fear scores were more prone to prefer epidural anesthesia and birth by CS compared to students with lower levels of fear [2, 6].

From studies focusing on fear of childbirth among pregnant women, we have learned that fear of childbirth can be associated with having postponed pregnancy or considered termination of pregnancy [12]. This observation is in line with behavioral models of anxiety, where avoidance of feared stimuli plays a central role [13]. If a consequence of fearing birth is to avoid pregnancy, there is a risk that some of the women who experience high levels of preconception fear may never venture to become pregnant.

Given the limited number of studies published within this area, a qualitative approach offering a description of women’s experiences of fearing childbirth before a first pregnancy could make a particularly important contribution to the field. To date, little is known about the experiences of these women.

The aim of this qualitative study was thus to give voice to the experiences of this often-neglected group of women. Using reflexive thematic analyses of interviews with women who wish to start a family but are too afraid to give birth, we contribute to a broader perspective on women’s experiences of living with fear of childbirth. We also sought to explore the experiences of and the need for health care support for these women. We believe that the present study will make a valuable contribution to the existing research on preconception fear of childbirth. In addition, this study may inform further research on this important topic and empower and support health care practitioners and policymakers to improve the support offered to women experiencing preconception fear of childbirth.

## Methods

### Design

In this qualitative study we conducted semi-structured interviews with nine non-pregnant women who had never given birth and were hesitant to become pregnant due to fear of childbirth. The study was part of a larger project that explored the experiences of fear of childbirth in non-pregnant women, both women who had the experience of pregnancy and childbirth and those who had not. As delineated by Braun and Clarke [14], reflexive thematic analysis is a suitable method to address research questions relating to contextually lived experiences, subjective perceptions, behaviors, and needs of particular groups in particular contexts. Given the similarity to our intentions, we used their framework for analysis, emphasizing an experiential, inductive, and semantic orientation.

Ethical approval was obtained from the national Ethical Review Agency February 6, 2020 (Dnr: 2019-06398).

### Recruitment

Women were recruited by calls for participants in social media (February 18–24, 2020). If interested, they could click on a link to an online registration form where they received further information about the study and consented to leave background and contact information. Background data included age, marital status, country of birth, level of education, and size of their hometown. To ensure we only included women in our target group, we also asked control questions regarding ongoing pregnancy (yes/no), previous childbirth (yes/no), wish to give birth in the future (yes/no), and preferred birth mode (vaginal/CS). These data were collected using the online survey software Qualtrics (Qualtrics; Provo, UT). Of the 44 women who registered their interest to participate, 21 were selected based on purposive sampling with the intent of having a broad representation of the different background characteristics in our sample. These women were contacted by email and/or telephone and invited to participate in an interview. They were informed about the study and encouraged to ask questions.

### Participants

In the end, nine women, 23–40 years of age, accepted the invitation and were interviewed. Seven were born in Sweden and two were born in another country (one in a Latin American country and one in an Eastern European country). Two participants were not in a relationship, and seven were living with a partner. Five lived in a relatively large city area, two in a medium-sized town, and three in a small town or rural area. Their level of education varied from high school to university level. Of the nine participants, four stated that they would prefer a vaginal birth and five would prefer a CS if they decided to have children.

### Data collection

Interviews were conducted in February and March 2020. Time and location for each interview were decided in dialogue with the participant based on their convenience. One interview was conducted by online video call, five by telephone, and three face-to-face. Each interview lasted between 45 and 90 min and was recorded using a Dictaphone. The interviews all began with a recorded verbal consent from the participant.

The interviews were semi-structured and followed an interview guide developed by the authors. The questions were pilot tested once, which resulted in some questions being revised to encourage detailed responses. The questions had three main areas of focus: participants’ descriptions of their fear and how it affected their lives; their need and wish for support; and their experiences of weighing their fear against their desire to have children.

According to the interviewer, all participants seemed relaxed during the interviews and spoke openly about their situation. There were no evident differences between interviewees in this regard whether interviews were conducted face-to-face or by other means. Instead, participants appeared to appreciate the opportunity to choose whether to meet in person, use a video call, or conceal their face by talking on the phone. Many participants spontaneously expressed their gratitude for having the opportunity to talk about their situation and that their experiences were taken seriously.

The interviewer transcribed the interviews continuously. After six interviews were transcribed, the material started to show signs of data saturation (i.e., similar content was found in most interviews). It was concluded that the nine already booked interviews were likely to be enough to reach saturation, so additional participants were not sought. The last three interviews confirmed this decision, as new interviews no longer provided new information.

### Data analysis

For data analysis, we used reflexive thematic analysis as described by Braun et al. [15]. With an inductive approach and focus on semantic themes, we strived for a rich overall description of the data set, close to the semantic content expressed by our participants. Therefore, we chose to take an experiential approach to thematic analysis, viewing the participants’ verbal descriptions as their true subjective and contextually-situated experiences [14].

Familiarization with data started during transcription and continued in repeated readings of the transcripts. After the initial manual coding of data, one of the authors drew a first thematic map that was further refined in dialogue between the authors. Potential themes were reviewed in relation to the codes, the data extracts, the other themes, and the study aim until the thematic map was found to be satisfactory and the themes could be defined. The themes were then discussed and refined once more to clarify the essence of each theme and sub-theme and to enhance depth and reflexivity. These final themes were named and checked against the codes and data extracts once more.

### Authors’ perspectives and reflexivity

The first author is a licensed psychologist and the last author a licensed midwife. Both are active researchers within the field of fear of childbirth. The second author, who conducted the interviews, was at the time a master’s student in clinical psychology. None of the researchers had any previous relationship with the participants.

With the central role given to researcher subjectivity and reflexivity in reflexive thematic analysis, we are grateful for our backgrounds in midwifery and psychology. It is our experience that this diversity helps us widen our perspectives and deepen our understanding of women’s experiences of fear of childbirth. To boost creativity and enhance our collective reflexivity, we found it central to engage in joint discussions during the latter parts of the analytic work.

As researchers, we were well acquainted with the literature on fear of childbirth, including its narrow focus on fear experienced during pregnancy. With this work, we had no other agenda than to broaden this focus by presenting a nuanced and rich interpretation of any experiences of fear of childbirth expressed by non-pregnant women.

## Results

**Fig. 1:**
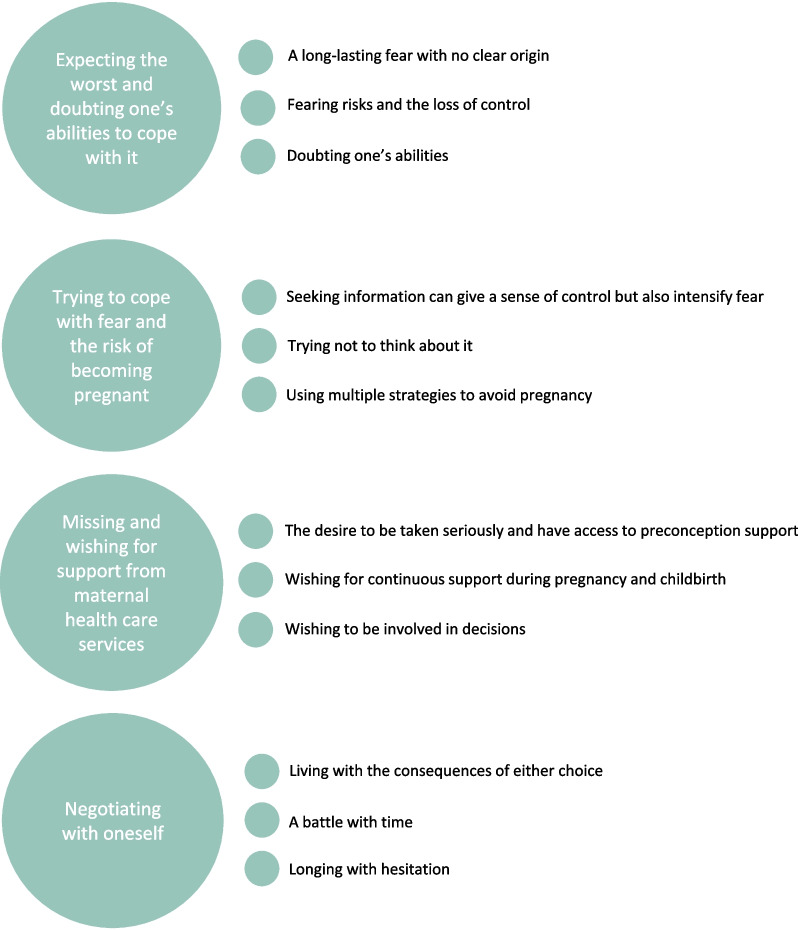
Themes and subthemes that describe the experiences of women expressing preconception fear of childbirth

All participants in this sample stated that they wanted to have children but were very afraid of giving birth. Four main themes that describe their experiences were identified (see Fig. 1): (1) Expecting the worst and doubting one’s abilities to cope with it; (2) Trying to cope with fear and the risk of becoming pregnant; (3) Missing and wishing for support from maternal health care services; and (4) Negotiating with oneself.

### Expecting the worst and doubting one’s abilities to cope with it

#### A long-lasting fear with no clear origin

These women described that their fear had existed as long as they could remember or that it had emerged when they were young adults. For most, the fear had no clear origin, but some had been influenced by negative birth stories from others. Although they were not explicitly asked about other mental health issues, a few of the participants revealed general symptoms of depression and/or anxiety, which might also have predisposed them to experience fear or anxiety related to childbirth.

#### Fearing risks and the loss of control

The idea of pregnancy and childbirth was perceived as the total loss of control. The participants envisioned chaos and trauma, including unbearable pain, panicking, or losing the child’s or one’s own life. This uncontrollable situation raised feelings of panic where death felt like the only way out:

The birth—you have no idea. It feels like playing Russian roulette. What will happen? Nobody knows!

#### Doubting one’s abilities

The women felt insecure about their ability to give birth and face all imaginable risks. Many compared themselves with others and felt bad as they did not dare give birth, while others seemed to do it without hesitation.

The participants described thoughts about being too sensitive to pain or too vulnerable or fragile to give birth. Previous experiences of pain such as menstruation, abortion, or vulvar pain made some women anxious about how they would cope with the more severe pain that they imagined they would experience during birth. The participants also doubted their mental abilities to handle birth:

I can trust my body, but I don’t think I trust my mind–am I able to handle what might happen? What do I do if I end up in this spiral and can’t break it? [...] If I just want to leave my body and I regret everything. That's my greatest fear.

### Trying to cope with fear and the risk of becoming pregnant

#### Seeking information can give a sense of control but also intensify fear

Many women tried to prepare and deal with their fear by seeking information, mainly on the internet and in social media. On the one hand, acquiring information gave them a sense of control. On the other hand, information could also lead to increased fear. Nevertheless, many found it difficult to stop seeking information, as they had few other strategies to use:

I don’t know, you usually say the more you know the better. But in this case, I think maybe I should watch and read less. Because today [...] we can read about anything and watch anything. I think that's what actually triggers this feeling the most.

#### Trying not to think about it

A central strategy to master fear was to make efforts to avoid thoughts about pregnancy and childbirth, using strategies such as thought suppression, avoidance of information, and not talking about these issues.

[I] repress it and don’t talk about it. Even my mom, I’m very close to my mom and she usually talks to me a lot and asks, and I just switch off completely. I kind of ban the topic.

#### Using multiple strategies to avoid pregnancy

The women used different strategies to avoid an immediate pregnancy (e.g., avoiding unprotected sex, choosing a same-sex partner, using emergency contraceptive pills, or even considering sterilization). Thinking about the possibility of terminating a pregnancy was calming to most of the participants, although the thought also made them sad as they had a strong desire to have children. The few women who had terminated a pregnancy due to fear described delayed responses of grief and emotional pain.

### Missing and wishing for support from maternal health care services

#### The desire to be taken seriously and have access to preconception support

The women shared the experience that others did not take their fears seriously. When professionals and people around them made attempts to normalize, reassure and encourage, they often felt that their concerns were being ignored and their fear minimized. This made them feel less than a “real” woman.

I believe that if I had received help and support now, before I get pregnant, to just talk and not be belittled in my fear, then maybe I would have dared get pregnant.

The participants did not know of any place where they could seek professional support for pregnancy concerns when not actually pregnant. They thought their problem would not be prioritized and were not sure if health care workers knew about these issues. They felt that it might feel less ridiculous and more natural to seek help if they were actually pregnant:

As there is nowhere to turn, I reason just like everyone says–the day I want to try to get pregnant or fall pregnant, I have to deal with the problem or the fear and see what help I can get.

The few women who had sought help had been well treated but felt vulnerable and it had not made the decision about becoming pregnant any easier.

In the interviews, the participants expressed a wish that health care support should be available for women in their position. They wished there was a health care facility they could turn to for both psychological support and an opportunity to talk to a midwife or doctor about childbirth. With earlier support, perhaps already when seeking contraceptive advice, the women believed that the decision to become pregnant might be easier and pregnancy less anxious:

I wish the help already existed. [...] The same kind of help but earlier–before you get pregnant and plan the whole process.

#### Wishing for continuous support during pregnancy and childbirth

The women further doubted their chances of receiving satisfactory support during pregnancy and childbirth. This distrust in maternity health care services was a major reason for avoiding pregnancy:

It would have felt so different if you knew you would be met with open arms by the health care system. Not just being another number but knowing that you really will be seen and that the care you’ll receive is 110 percent.

Concerns regarding insufficient health care resources were common, and some participants were intimidated by media stories about how health care can fail. Other participants trusted the health care services in general but still doubted being acknowledged as someone needing care in this vulnerable situation. Many stated that they might have dared become pregnant if they knew for sure that they would receive continuous support during pregnancy and birth.

#### Wishing to be involved in decisions

The importance of being involved in decisions was emphasized, most importantly in decisions about one’s birth mode. Some women found that the thought of a planned CS gave some sense of control, so they expressed a preference for birth by CS. They expected this desire to be met with opposition and resistance and were afraid they would be persuaded or forced to choose a vaginal birth. For these women, finding information about CS birth could be used to build one’s arguments for a CS birth in the event of a pregnancy. Several thought they would be more open to try giving birth vaginally if CS were an option if the birth situation got unbearable. However, they were not sure such a promise would be kept:

If I knew, that if I say that I want to try to give birth vaginally and then say that I don’t, I’d get a caesarean. To be understood and listened to as a woman. [...] Then I guess it would have felt okay.

### Negotiating with oneself

#### Living with the consequences of either choice

When deciding whether to dare become pregnant and give birth, the women focused on the potential negative consequences of both alternatives. By exposing themselves to the risks of giving birth, the women imagined they would have to accept being injured for life, resulting in a ruined sex life and poor mental health. By avoiding pregnancy, the women imagined their life would be lonely and filled with grief:

If you have to live without a sex life, being incontinent and barely able to move, that wouldn’t be a future that I’d want. But then you’d have a child or two instead.

Another woman said:

I’ve always imagined having a family, and I still want that. When everyone gathers for Christmas at home with their families, my place will be empty. Is that what it will be like for the rest of my life?

Deciding not to have children could also affect relationships with a partner. The women revealed fear of rejection and actual experiences of having been left because of fearing birth. Fear of pregnancy and childbirth could also impede the search for a partner or prevent commitment in relationships. If a partner definitely wanted to have children, these women felt pressured to decide whether to go ahead and have children despite their fear or to end the relationship. Women living alone also struggled with deciding whether to have children on their own. Not having children due to fear was further perceived as a risk of societal exclusion. Some had considered adoption or surrogacy as possible ways to become a parent without having to give birth themselves, but they found the thought of exposing another woman to childbirth objectionable.

#### A battle with time

When these women were younger, fear was often peripheral. As they aged or formed a stable relationship, the idea of children became real and fear grew stronger. However, some found that their fear decreased as they aged.

I feel stressed [...] that I have to do it as soon as possible, that I’m starting to get old. [...] I don’t feel that I’m old-old, but that my biological clock is ticking.

The fact that the risks of giving birth increase with age evoked frightening images of waiting too long or dying during childbirth. As the decision about whether to have children was often postponed, some participants expressed that there was a level of stress about not having enough time to have more than one child.

I understand that the older I get, the more I can legitimize my fear, [...] because the risks increase with age. And can you even become pregnant? So, it is incredibly stressful to feel that you are running out of time. [...] It’s almost worse than the fear.

Despite having a desire for children soon, many of the women alternated between deciding to get pregnant and deciding to postpone it again. They expressed strong concerns that the fears would grow stronger over time and that they might be waiting for a chance that will never come–a window of opportunity when it feels easier, suits better, or the desire for a child suddenly becomes stronger than the fear. It was difficult to see others form a family while they did not. In addition, some thought they would probably have started building a family by now if they had not been afraid.

#### Longing with hesitation

The women in this study all wanted to have children. While some described their desire as strong, others indicated that having children had not been that important to them. When comparing themselves to others who seemed more committed to having children, they found it difficult to differentiate whether their own doubts originated in fear of pregnancy and childbirth or whether it was because they did not want children to the same extent. They also perceived that others questioned whether their desire to have children was strong enough, which gave further fuel to their own doubts. They reasoned that perhaps they did not deserve to have a child.

Don’t I want to do it, or is it my anxiety? Am I avoiding it because it is too frightening? What is what?

Those who had challenged their fear and started trying to become pregnant described ambivalent emotions when finding out they were not pregnant–an immediate relief followed by feelings of guilt and sadness. In general, the women found it easier to allow themselves to long for children when the idea of building a family lay in a distant future or during periods when they knew they were not or could not become pregnant:

Once you realize that you aren’t pregnant, then it is perfectly okay to think about all this child stuff again, because then I know one hundred percent that I am not pregnant. So, then you can start googling children’s stuff.

Some women described that part of them wished they did not want to have children or were unable to become pregnant as at least that would reduce the pressure of having to choose.

## Discussion

The aim of this qualitative study was to give voice to the experiences of women who want to start a family but who do not dare become pregnant due to fear of childbirth. In addition, this study explored their need and desire for health care support. Using reflexive thematic analysis of nine interviews, we identified four main themes that described the experiences of these women.

The first theme, *Expecting the worst and doubting one’s abilities to cope with it*, indicated that the women in our sample had lived with their fears for a long time. As with pregnant women fearing birth [11, 12, 16, 17] and previous studies of non-pregnant women [6, 7], they viewed childbirth as inherently risky and uncontrollable and doubted their own capacity to deal with the situation.

The second theme, *Trying to cope with fear and the risk of becoming pregnant*, described how the women tried to deal with their feelings by trying not to think about it and by seeking information. They avoided becoming pregnant and thought about alternative ways of building a family. Although non-pregnant samples have seldom been studied, pregnant women have previously reported similar coping strategies [18].

Although the women in this study expressed many aspects of fear of childbirth in similar ways as pregnant women, the third and fourth themes seem more specific to this particular group and therefore add a new perspective to the existing knowledge regarding fear of childbirth. In the third theme, *Missing and wishing for support from maternal care services*, the women reported having very limited opportunities to receive support from maternal care services before becoming pregnant. They asked for a dedicated health care service that they could turn to for both psychological support and an open and honest dialogue concerning possible risks and birth options in the event of pregnancy and childbirth. Participants disclosed having terminated a pregnancy because of their fear of childbirth. A decision such as this may have been avoided if support to help deal with the fear had been available before pregnancy or in early pregnancy.

The limited resources allocated to support this group can be confirmed from the clinical viewpoint. Although Swedish maternal care services supporting pregnant women with fear of childbirth are relatively well organized [19], support for non-pregnant women suffering from fear of childbirth is clearly in need of improvement. On isolated occasions, fearful women may receive support in advance of becoming pregnant, but often women who fear childbirth are left to manage on their own. However, as maternal care services in Sweden also meet women in preconception care, this seems to be the most natural place to seek and receive support also for non-pregnant women who fear childbirth.

When thinking about a future pregnancy, the women in this study emphasized a desire to receive continuous support. As intrapartum care with a known midwife has been associated with a more positive birth experience [20], this desire seems reasonable.

Instead of being dismissed or met with opposition, the women asked to have their experiences taken seriously and wanted to be involved in making important decisions. As has been reported by others [7], involvement in decisions about birth mode was particularly important. Unfortunately, several women in our study expressed distrust of maternal care services and were not convinced they would receive the support they needed in the event of a future pregnancy. Previous research has shown that women rely heavily on support from maternal care services in difficult reproductive situations and that the perception of this support is largely determined by experiences of personal autonomy [21]. Like the women in our sample, Clarke et al. [21] argue for including women as active agents and ensuring transparency in the services offered to support a true reproductive choice. If women were assured they would be involved and supported in important decisions, more women might feel safe enough to dare become pregnant.

Unlike pregnant women, the women in this sample still had the choice to keep avoiding pregnancy. For decades, behavioral psychologists have given avoidance a central role in theoretical and empirical models explaining the etiology and maintenance of anxiety [13]. As expressed in the fourth theme, *Negotiating with oneself*, the women found themselves trapped in a state of ongoing negotiation with themselves, wondering whether they would succumb to their fears or dare challenge them and embrace pregnancy. They felt they had to make the decision on their own and feared they would regret whatever decision they made. The participants reported that if they did decide one way or the other, it was often retracted, leading to endless, tedious, and stressful rumination. The inability to come to a decision and commit to it was accompanied with feelings of sadness, shame, and repressed longing. With regard to reproductive decision-making, Miller [22] concludes that it is possible and probably common that women have both positive and negative childbearing desires simultaneously. Similar ideas have been proposed by Bernardi et al. [23], who present a typology with six categories of fertility intentions, of which only two describe certain intentions (“definitively *yes”* and “definitively *no”*). The remaining four categories are characterized by uncertainty but differ qualitatively with regard to the sources of this uncertainty, how it is expressed, and how it is dealt with. Some couples have a strong desire to have children but not in the near future (“far intentions”). For others, uncertainty is mainly linked to the internal desire of having or not having a child (or another child) (“indifferent intentions” or “ambivalent intentions”). This can be contrasted with the category of “contingent intentions,” where the desire to have a child is strong, but perceived external obstacles, often beyond the individual’s control, result in uncertainty. The women in our sample are not easily characterized in any of these six categories. As most of them clearly expressed a strong desire to have children but perceived obstacles preventing them from proceeding, their intentions could probably be placed in the category of contingent intentions. However, untypical for this category, their uncertainty originated not only from external sources but also from internal experiences of fear, worry, and anxiety. Presently, many women who are childless due to medical conditions can be offered help and support from existing health care services. When the desire to have children is impeded because of psychological factors such as fear or anxiety, women and their partners still lack adequate support.

### Implications

From a clinical perspective, treating fear of childbirth during pregnancy can be challenging, and we are still short of evidence-based interventions [24, 25]. As pregnancies normally only last nine months (sometimes less), there is only a short time when interventions can take place. It is also rather common to meet women who are determined to give birth by CS and who are not interested in getting support for a vaginal birth. In this sense, it might be beneficial to offer interventions to reduce fear of childbirth before a woman’s first pregnancy.

Previous research has shown that fear during pregnancy is related to more negative birth experiences [26–28], which in turn is related to fear in a subsequent pregnancy [29–31]. Previous studies have also shown that fear of childbirth during pregnancy is associated with extra antenatal care visits, more hours of sick-leave, longer stays at the maternity ward, and more postpartum care visits [32]. If reasonable support can be given at an early stage, there might be a better chance of breaking the negative chain of events and of having a positive impact on a woman’s reproductive life from the start. It might even be possible to reduce women’s need for support during pregnancy and the perinatal costs shown by Nieminen et al. [32]. Naturally, the limited resources for maternal health care have to be used wisely and decisions need to be made about what to prioritize. By not providing these women with the support they need at an early stage, care interventions are merely postponed, perhaps to a less favorable time, when fear has increased, negative expectations are already established, and clinical interventions are limited by time constraints. The postponing of interventions also means leaving these women alone in their negative thoughts and, at worst, leading to the decision to give up on their dream to have children.

### Limitations

The findings need to be considered in the light of some study limitations. Above all, this study is built on experiences from a rather small study sample. Although originally part of a larger project that included women who had given birth before, we decided to split the sample in two in order to focus on the specific experiences of women suffering from fear of childbirth before their first pregnancy. Within this specific sample, care has been taken to ensure heterogeneity in terms of age, education, civil status, and place of residence.

### Conclusion

In conclusion, women’s experiences of fear of childbirth before a first pregnancy seem similar to the experiences reported by pregnant women. However, unlike pregnant women, the women in this group lack support from maternal care services. Their experiences of not having a dedicated health care service to turn to is reflected in maternal care policies. In addition, their desire for both psychological support and an open dialogue regarding their options in the event of pregnancy and childbirth should be taken seriously. Often, these women are left on their own as they try to decide whether to dare become pregnant. Offering them the support they ask for might not be a waste of time and resources but a responsible and wise investment.

## Data Availability

The datasets generated during the current study are not publicly available due to the risk of comprising individual privacy.

## Abbreviation

CS: Caesarean section
